# How Focusing on Superordinate Goals Motivates Broad, Long-Term Goal Pursuit: A Theoretical Perspective

**DOI:** 10.3389/fpsyg.2018.01879

**Published:** 2018-10-02

**Authors:** Bettina Höchli, Adrian Brügger, Claude Messner

**Affiliations:** Department of Consumer Behavior, Institute of Marketing and Management, University of Bern, Bern, Switzerland

**Keywords:** superordinate goal, goal hierarchy, goal abstraction, goal pursuit, long-term

## Abstract

Goal-setting theory states that challenging, specific, and concrete goals (i.e., subordinate goals) are powerful motivators and boost performance in goal pursuit more than vague or abstract goals (i.e., superordinate goals). Goal-setting theory predominantly focuses on single, short-term goals and less on broad, long-term challenges. This review article extends goal-setting theory and argues that superordinate goals also fulfill a crucial role in motivating behavior, particularly when addressing broad, long-term challenges. The purpose of this article is to shed light on the benefits of superordinate goals, which have received less attention in research, and to show theoretically that people pursue long-term goals more successfully when they focus on subordinate as well as superordinate goals than when they focus on either subordinate or superordinate goals alone.

## Introduction

Pursuing and achieving goals is difficult. This is not only documented in the academic literature (Ryan, 2012), but is also evident from the large number of self-help books available. Amazon (2018) lists over 120,000 books in the motivational self-help category, which suggests that many people seek help with achieving goals. When struggling with goals such as “lose weight” or “exercise more”, goal-setting theory is one of the most prominent approaches to improve performance in goal pursuit (Locke and Latham, 2013).

Goal-setting theory examines how setting a goal influences subsequent performance in pursuit of that goal. It focuses mostly on the specificity and performance level of goals (Locke and Latham, 2013). Goal-setting theory shows across hundreds of studies that challenging, specific, and concrete goals are powerful motivators and boost success in goal pursuit more than do vague and abstract goals (e.g., Locke and Latham, 1990, 2002, 2013). For example, the specific goal “lose 10 pounds in 2 months” should be more successfully achieved than the vague goal “lose weight” (Locke and Latham, 2002).

A crucial limitation of the research conducted within this paradigm is that studies typically examine the effect of setting a specific, concrete, and challenging goal versus an abstract, vague goal on the performance of a single task. In contrast to this relatively narrow focus, many of today’s social, environmental, and economic challenges hinge on broad, long-term goal-pursuit. To address a health problem such as obesity requires more than to “lose 10 pounds” once; it requires a continued healthy diet as well as regular exercise. Similarly, “creating less garbage” requires increased recycling and reduced consumption in the long run. And a businessperson whose goal is to increase profits relies on, among other things, continued employee motivation. When facing such long-term challenges, goal-setting theory suggests the goal-setter should subdivide abstract, long-term goals—superordinate goals—into specific, concrete short-term goals—subordinate goals—in order to enhance both motivation and performance (Steel and König, 2006; Sun and Frese, 2013). However, in order to successfully address broad, long-term challenges, besides achieving single steps, people need to face and overcome various obstacles: sustaining motivation over the long term, resisting the pull of competing goals and temptations, overcoming compensation effects, being resilient in the face of setbacks and failures, and more (e.g., Rothman et al., 2004). In light of such broad, long-term challenges, focusing solely on subordinate goals may not be the best solution (Ordóñez et al., 2009).

One approach that might help to overcome these difficulties is to focus more on superordinate goals. This article summarizes theoretical and empirical evidence that superordinate goals and subordinate goals each contribute to successful goal pursuit through distinct processes, and work best when combined. While the effects of subordinate goals are well examined in the context of goal setting theory, relatively little is known about how superordinate goals influence goal pursuit and how they interact with subordinate goals (Day and Unsworth, 2013).

This article first describes the goal hierarchy and how subordinate and superordinate goals differ. Then, focusing on superordinate goals and their potential benefits, the article outlines three characteristics of superordinate goals: Superordinate goals are identity-based, have an extended temporal perspective, and entail a broad scope of contexts. For each characteristic, the article describes several self-regulatory processes by which superordinate goals foster goal pursuit. Based on the discussed benefits of superordinate goals and the advantages of subordinate goals identified by previous research, the article highlights the fact that subordinate and superordinate goals are by no means exclusive, but on the contrary, are beneficial when combined. The article concludes by pointing out relevant open issues for future research.

## Goal Hierarchy

A goal is a mental representation of a desired end state that a person is committed to approaching or avoiding. Goals can differ in various characteristics (Moskowitz, 2012) which can influence subsequent motivation and performance (Locke and Latham, 2002; Moskowitz, 2012). One of the most fundamental characteristics of a goal is its level of abstraction (Fujita and MacGregor, 2012). Consider the goals “be in good physical shape,” “do 40 push-ups on Wednesday afternoon,” “eat a healthy diet,” or “be healthy.” These goals are all related to being healthy, but they differ in their level of abstraction (Carver and Scheier, 2001).

**FIGURE 1:**
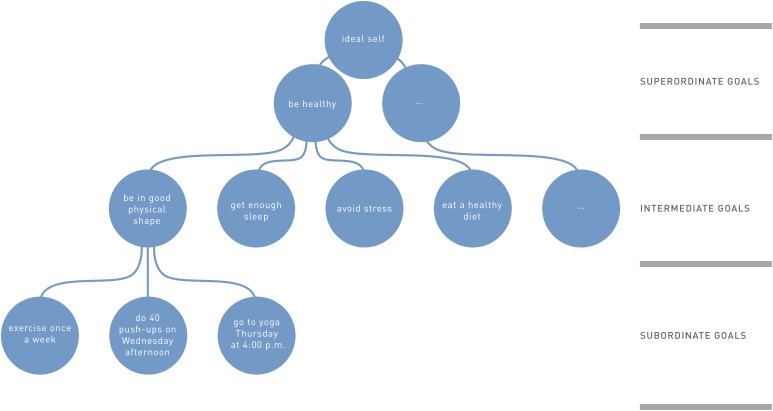
Schematic representation of goals at three levels of abstraction (adapted from Carver and Scheier, 2001, with permission).

The lowest level of abstraction contains subordinate goals (Carver and Scheier, 2001) (see **Figure 1**). They define precisely what to do and how to do it. By taking into account environmental affordances and constraints, they specify concretely how goals one step up in the hierarchy—that is, intermediate goals—can be achieved (Boekaerts et al., 2006). For example, the subordinate goals to “go to yoga classes on Thursday at 4:00 p.m.” or “do 40 push-ups on Wednesday afternoon” could help achieve the intermediate goal to “be in good physical shape.”

Intermediate goals then provide a general course of action that is bound to a certain behavioral context; for example, “run a marathon” or “eat a healthy diet” (Carver and Scheier, 2001). Multiple intermediate goals across different behavioral contexts in turn help achieve goals even higher up in the hierarchy—that is, superordinate goals. For example, the intermediate goals to “be in good physical shape,” “get enough sleep,” “avoid stress,” and “eat a healthy diet” all contribute to the superordinate goal to “be healthy.”

Superordinate goals are at the highest level of the goal hierarchy. They refer to idealized conceptualizations of one’s self, one’s relationships, or the society one is part of. These superordinate goals reflect what is (not) important to a person (Boekaerts et al., 2006). As such, superordinate goals are very similar to values. This is obvious, for example, in Schwartz’s (1992) conceptualization and measurement of values. Schwartz holds that “the primary content aspect of a value is the type of goal or motivational concern that it expresses” (p. 4) and directly asks participants to indicate the importance of different values “as a guiding principle” in their lives (Schwartz, 1992). This demonstrates the close alignment between superordinate goals and values. Even though values are not included in the hierarchical model, they are a related construct (Rokeach, 1973; Emmons, 1989) and can theoretically be considered to be one step up from superordinate goals (Boekaerts et al., 2006; Masuda et al., 2010). As the transition is fluent, the terms are often used interchangeably (Schwartz, 1992; Masuda et al., 2010).

When considering all three hierarchical levels, the interconnection of goals at different levels becomes apparent: Superordinate goals determine more concrete goals at the intermediate level, and intermediate goals in turn determine goals at the subordinate level. Thus, these goals can be placed into a hierarchy of levels of abstraction (e.g., Carver and Scheier, 2001; Kruglanski et al., 2002) in which subordinate goals are the means to reach a higher order goal (see **Figure 1**).

The goals within the hierarchy are interconnected and as a consequence can activate (or inhibit) each other: Focusing on a superordinate goal activates the connected subordinate goals or means (top-down activation, Kruglanski et al., 2002). Similarly, engaging in a behavior can bring to mind the connected superordinate goal (bottom-up activation, Shah and Kruglanski, 2003). When a person is exposed to or engages in a certain behavior it can automatically activate a connected higher order goal and thus the activation of goals may happen outside one’s awareness (Kruglanski et al., 2002; Shah and Kruglanski, 2003).

The connections among the goals in **Figure 1** represent a diversion from similar theories based on a hierarchical structure such as Action Identification Theory or Construal Level Theory.

Action Identification theory (AIT) holds that any action (e.g., preparing a meal) can be identified in different ways, ranging from concrete, specifying how the action is performed (e.g., cutting an onion, lifting a knife) to abstract, signifying why or to what effect the action is performed (e.g., to spend time with friends, to get to know a new culture) (Vallacher and Wegner, 1987). This overlaps to a certain degree with a goal hierarchical approach, in that superordinate goals may be viewed as identifying an act at a high level of abstraction and subordinate goals as identifying an act at a low level of abstraction. However, unlike a goal hierarchy with several interconnected goals, in action identification theory an action is identified either in a concrete or an abstract way and there is no interconnection or mutual activation of concrete or abstract identifications. Furthermore, action identification theory posits a natural tendency for people to drift upward to higher levels of identification with increasing familiarity and expertise: When engaging in a behavior that is new, challenging or difficult, people identify it on a concrete level and focus on the specific steps they have to conduct. As the behavior becomes less challenging they identify the action at a more abstract level and continue with this more abstract identification as long as they can successfully maintain it (Carver and Scheier, 2001). In contrast, to the best of our knowledge there is no such tendency to drift upward in a goal hierarchy with increasing simplicity, familiarity, or expertise.

Furthermore, the differentiation of goals according to their level of abstraction overlaps to some extent with the difference between concrete and abstract mindsets described in Construal Level Theory (CLT, e.g., Trope and Liberman, 2010). CLT argues that people can think about the same things (e.g., actions, events, goals) in concrete or abstract terms. According to CLT, the psychological distance at which people mentally represent things elicits different mindsets: The more psychologically proximal things are (e.g., happening to me, happening soon), the more concretely they are represented, which results in a focus on specific, concrete features of a given piece of information. By contrast, with increasing distance (e.g., happening to strangers, happening in the distant future) people think more abstractly about the same things and consider information in a more abstract fashion. At first glance, this suggests a similarity between focusing on a superordinate goal and adopting an abstract mindset as well as between focusing on a subordinate goal and adopting a concrete mindset.

However, there are some crucial differences between the CLT framework and the perspective presented here. First, the abstract and concrete mindsets in CLT are mutually exclusive: a person adopts either an abstract or a concrete mindset. In contrast, goals at different levels of abstraction are not mutually exclusive, but complementary (e.g., Carver and Scheier, 2001; Kruglanski et al., 2002).

Second, CLT and the goal hierarchy address different types of abstraction. CLT is interested in how people generally perceive the world and how they make decisions depending on whether they are in a concrete or abstract mindset. Thus, in CLT studies the level of abstraction of the goal itself does not vary: the goal itself remains the same, and only the way people perceive the information they consider necessary to pursue the goal changes (Liberman and Trope, 1998; Moskowitz, 2012). By contrast, our goal hierarchical approach is interested in the level of abstraction of the goals themselves and how goals at different levels of abstraction influence goal pursuit (Moskowitz, 2012; Burgoon et al., 2013).

Third, the accessibility of mindsets dissipates gradually over time – as does other cognitive material (Freitas et al., 2004). In contrast, goals are expected to remain accessible until they are satisfied (Zeigarnik, 1927; Bargh et al., 2001). This conceptual distinction between goals and mindsets is crucial with regard to the processes that subordinate and superordinate goals can trigger, especially over the course of time (for a similar reasoning, see Freitas et al., 2004).

A final conceptual distinction concerns superordinate goals and vague goals. Although goal-setting theory often contrasts concrete, specific goals with abstract, vague goals such as “do your best” (e.g., Locke and Latham, 2006), a superordinate goal is abstract but not necessarily vague, and a subordinate goal is concrete but not necessarily specific. Even though superordinate goals are by definition less specific than intermediate and subordinate goals, goals at all levels of abstraction can be formulated to be more or less specific or vague. For example, a subordinate goal can be formulated to be specific (e.g., “do 40 push-ups on Saturday afternoon”) or to be vague (e.g., “exercise more on the weekend”). Similarly, superordinate goals can be much more specific than just an appeal to make an effort (as in “do your best”, or “work hard”). As the next section will show in more detail, superordinate goals can provide a sense of direction, contain information about the value of the goal, place subordinate goals into a broader context, and fuel a person’s motivation to work toward their goal.

## Characteristics and Goal-Relevant Processes of Superordinate Goals

**FIGURE 2:**
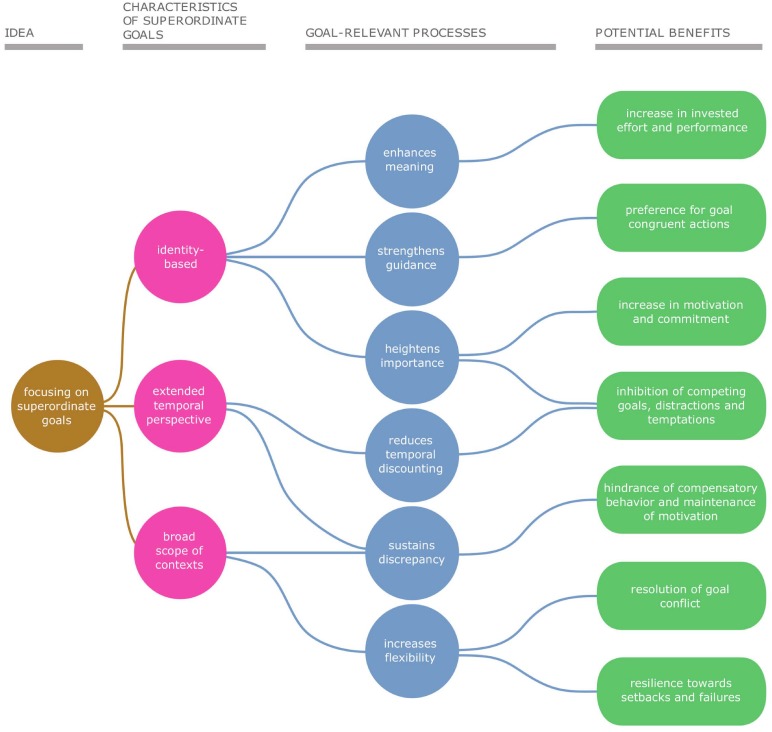
Overview of the three characteristics of superordinate goals and the related processes that foster successful goal pursuit.

Setting goals at different levels of abstraction can affect goal pursuit through various distinct processes. While goal-setting theory has widely studied the processes by which subordinate goals increase performance (Locke and Latham, 2013), less is known about the processes by which superordinate goals can increase motivation and foster goal pursuit. To address this gap, this article outlines three characteristics of superordinate goals and the resulting processes by which superordinate goals can promote broad, long-term goal pursuit. **Figure 2** gives an overview of these characteristics.

### Superordinate Goals Are Identity-Based

The first characteristic of superordinate goals which is relevant for long-term goal pursuit is that they are closely connected to an idealized conceptualization of a person’s self and are based on their identity (Oyserman and James, 2011). Superordinate goals describe how a person wants to be and reflect what is (not) important to a person (Boekaerts et al., 2006). Based on this close relationship between superordinate goals and identity, it can be argued that focusing on identity-based superordinate goals fosters goal pursuit in the long run for at least three reasons: it provides and enhances meaning, it strengthens guidance, and it heightens goal importance (see **Figure 2**).

#### Enhanced Meaning

Superordinate goals represent and determine what people ultimately value and aspire to (Carver and Scheier, 2001). As such, they address why a person wants to engage in a certain behavior and provide a reason or meaning for a target behavior (Carver and Scheier, 2001). Focusing on a superordinate goal provides a reason why identity-related actions are important and thus leads to better performance on those actions (e.g., Sheldon and Elliot, 1999). For example, participants who think about why vitamin intake is important for them increased their vitamin intake over two weeks compared to participants who didn’t think about why it was important (Chatzisarantis et al., 2010). This effect can also be seen in the working context; in a cross-sectional study across different occupations, people report a positive relation between meaning in work and work engagement (Höge and Schnell, 2012). The “why” or meaning of the task can refer not only to personal benefits but also to benefits for others: Fundraising callers who read stories about why and how their job helps others increased job performance, namely the number of pledges earned and the amount of money raised, up to 1 month after the manipulation, compared to callers who didn’t receive information about why their work was important (Grant, 2008, study 1).

#### Strengthened Guidance

Focusing on superordinate goals not only increases performance but can also guide the choice of identity-congruent actions. Superordinate goals connect closely with personal values (Boekaerts et al., 2006), are relatively stable over time, and represent what a person ultimately hopes for (Carver and Scheier, 2001). As such, superordinate goals may guide the selection of actions and the evaluation of people and events (e.g., Schwartz, 1992; Verplanken and Holland, 2002). For example, participants who were primed with superordinate goals such as preserving nature, caring for future generations, or living in a healthy place put more weight on environmental factors in a consumer choice task and would thus choose a television set that was more favorable in environmental aspects, as compared with participants in the control condition (Verplanken and Holland, 2002, study 1). That superordinate goals guide the choice of goal-relevant actions is further reflected in research on identity-based motivation. When focusing on a superordinate goal, people interpret situations in ways that are congruent with their identity, and prefer identity-congruent to identity-incongruent actions. For example, participants indicated a greater desire to approach people as potential friends when those people were instrumental to the participant’s superordinate goal, compared to people who were merely associated with or irrelevant to the superordinate goal (Slotter and Gardner, 2011). As the choice of social relationships is a pathway to self-change, a desire to approach certain people can be seen a preference for identity-congruent actions.

Research on organizational “vision” ties in with this argument. On an organizational level, a vision is defined as a future image of the organization that relates to values and describes a future state that the organization wants to achieve, and thus provides guidance (Shamir et al., 1993; Berson et al., 2001; Stam et al., 2014). Relevant research mainly focuses on the positive influence of an organizational vision on performance and growth (Kirkpatrick and Locke, 1996; Lawson and Shen, 1998; Locke and Latham, 2006; Masuda et al., 2010). Having an organizational vision along with growth goals and self-efficacy predicts venture growth (6-year longitudinal study with small-venture entrepreneurs; Baum and Locke, 2004). Even though the role of visions is predominantly discussed in the context of organizations, the related insights should, to some extent, apply on a personal level (Stam et al., 2014). In that sense, a vision describes a concept of oneself in the future, reflects personal values, and describes desired outcomes, and thus aligns with the characteristics of self-concordant superordinate goals (Masuda et al., 2010; Locke and Latham, 2013; Stam et al., 2014). Research provides support for the potential benefits of a personal vision to goal pursuit. A challenging and vivid personal vision increases student’s commitment to their semester goals (Masuda et al., 2010). Consequently, research on personal visions supports the proposition that self-concordant superordinate goals function as antecedents of the described positive effects on broad, long-term goal pursuit.

#### Heightened Importance

A third reason why superordinate goals foster long-term goal pursuit concerns goal importance. The higher a goal is in the hierarchy, the more fundamental the goal is to the overriding sense of self. Goals at a higher level are intrinsically more important than those at lower levels (Carver and Scheier, 2001). Focusing on a superordinate goal can foster goal pursuit by increasing motivation and commitment and by helping to resist distractions and temptations.

Motivation – which activates, directs, and sustains goal-directed behavior – increases as a product of a goal’s importance and the expected likelihood of reaching a goal (e.g., Eccles and Wigfield, 2002). This motivational benefit of goal importance is also reflected in research on goal commitment (Klein et al., 1999). Goal commitment captures the pledging of oneself to a goal: Goals will not operate as intended without commitment (Klein et al., 2013). Similarly to motivation, the product of goal importance and the expectation of reaching the goal has been found to be significantly related to goal commitment (Klein et al., 1999). This implies that the importance of a goal is crucial to increase motivation and commitment, but is only effective when people believe that they can reach the goal. While the importance of superordinate goals is higher than of subordinate goals, subordinate goals can facilitate self-efficacy beliefs; that is, beliefs that one is capable of carrying out the steps required to achieve the intended effect (Bandura, 1997). Thereby, the expectation of reaching the goal increases (Klein et al., 2013). Thus, focusing on superordinate goals increases motivation and commitment, as long as a superordinate goal is combined with subordinate goals that define the steps to attain the superordinate goal.

Broad, long-term goal pursuit requires people to not only invest effort, but also to ignore distractions and resist temptations (Cavallo and Fitzsimons, 2012). As people normally pursue multiple goals and have limited resources, goals compete for resources. When considering numerous competing goals, a person has to prioritize which goal(s) to pursue, ultimately putting effort into some goals while setting aside those that represent a distraction or temptation (Fishbach and Ferguson, 2007). Goals with a high level of importance and commitment – that is, superordinate goals – are more likely to be prioritized over goals at a lower hierarchical level with a lower level of importance and commitment (Cavallo and Fitzsimons, 2012).

Another way in which focusing on a superordinate goal can help to more effectively pursue goals is by inhibiting alternative goals (Shah et al., 2002). Alternative goals can interfere with the current goal pursuit as they can be a distraction and pull away resources. Thus, the more people are able to inhibit alternative goals, the more persistent they are in their primary goal pursuit. Importantly, the extent to which people are able or willing to inhibit alternative goals depends on their commitment to a given goal: the stronger the commitment, the more its activation inhibits the accessibility of alternative goals (Shah et al., 2002). It follows that focusing on superordinate goals –and the corresponding high level of commitment they promote – can inhibit possible distractions and thus foster persistent goal pursuit.

### Superordinate Goals Entail an Extended Temporal Perspective

The second characteristic of superordinate goals is an extended temporal perspective. Firstly, that means that a goal is pursued over a longer time period. As a superordinate goal is linked to multiple subordinate goals, it requires multiple behaviors across multiple instances. For example, the superordinate goal to “be healthy” cannot be achieved by taking one yoga class, but requires multiple goal-consistent actions over time. Secondly, superordinate goals do not have a specific endpoint. It is difficult to determine a specific time when the goal to “be healthy” is or should be reached. The only temporal information the goal conveys is that it requires a longer rather than a shorter time (Fujita and MacGregor, 2012). An extended temporal perspective can foster broad, long-term goal pursuit in two ways: with reduced temporal discounting and sustained discrepancy (see **Figure 2**).

#### Reduced Temporal Discounting

Temporal discounting refers to the tendency to relinquish larger future rewards in favor of smaller immediate rewards (Urminsky and Zauberman, 2015). Common examples of temporal discounting include choosing a slice of chocolate cake over an apple, thereby favoring enjoyment in the present over long-term good health, or spending money now rather than saving for the future (Urminsky and Zauberman, 2015). This preference for the immediate over the distant outcome can undermine goal pursuit as it can induce a person to give in to short-term temptations at the cost of long-term goals. Focusing on superordinate goals can help to reduce temporal discounting through at least two mechanisms.

First, focusing on superordinate goals can reduce temporal discounting by construing the goal in an abstract and distant manner instead of a concrete manner (Urminsky and Zauberman, 2015). To illustrate, participants who responded to questions related to why they engaged in a certain action showed a reduced tendency to prefer an immediate over a delayed outcome across four purchasing scenarios compared to participants who answered questions about how they engaged in an action (Fujita et al., 2006, study 1). As focusing on a superordinate goal, and thereby adopting an extended temporal perspective, represents the future desired outcome in an abstract and distant manner, it follows that focusing on a superordinate goal can increase the preference for the distant compared to the immediate outcome.

Second, focusing on a superordinate goal can reduce temporal discounting as it strengthens the connection to one’s future ideal self. A superordinate goal reflects how and who people want to be in the future. The more people feel psychologically connected to their future self, the less they discount future monetary and non-monetary benefits (Bartels and Rips, 2010, studies 1 and 2). Furthermore, people who perceive a high connectedness to their future self through a manipulation task (reading passages highlighting stability versus changes in one’s future identity) demonstrate less temporal discounting; they require a smaller delay premium to wait for a gift card, are less likely to favor a less valuable gift card sooner over a more valuable gift card later, are more willing to wait to buy a computer that declines in price, and discount the value of money less than people who feel less connected to their future selves (Bartels and Urminsky, 2011, studies 1–5).

#### Sustained Discrepancy

The second way in which an extended temporal perspective may assist long-term goal pursuit has to do with sustained discrepancy between the status quo and the desired end point. During goal pursuit, this discrepancy fuels motivation. According to goal-setting theory as well as several other theories concerned with goal achievement and motivation (e.g., control theory, Carver and Scheier, 1982; self-discrepancy, Higgins, 1987; symbolic self-completion, Wicklund and Gollwitzer, 1982), people monitor where they stand in relation to their goals. People desire and work to decrease the discrepancy between their current state and their goal (Carver and Scheier, 2001). They persist with a certain behavior and inhibit competing goals until the discrepancy is reduced and the tension is alleviated (Lewin, 1936). Crucially, this implies that once a goal is achieved, the discrepancy, and thus the resulting motivational impulse, disappear (Kruglanski et al., 2002; Moskowitz, 2012).

Often, the tendency to relax one’s efforts after achieving a goal is useful as this makes limited resources available for other goals. However, this tendency can have detrimental effects, particularly in the context of broad, long-term goals that require many steps. When facing this kind of goal, relaxing after the achievement of a first step or subordinate goal hinders subsequent goal pursuit and the achievement of the superordinate goal. The achievement of the subordinate goal signals that the person has done what is necessary and they can stop pursuing that particular goal. Dieters who have successfully lost weight during the course of a diet may regain weight afterward (Lowe et al., 2001), and exercisers stop exercising. As such effects – often referred to as compensation effects – can arise due to dissolved discrepancy between the status quo and the desired end (e.g., Carver and Scheier, 2001; Dolan and Galizzi, 2015) the reverse should also hold; that is, sustaining the discrepancy should prevent these compensation effects.

As superordinate goals can hardly, if ever, be fully achieved, they sustain the discrepancy between the status quo and the desired end state and thus can weaken compensation effects and sustain motivation. In other words, when people pursue superordinate goals, achieving a subordinate goal only signals partial fulfillment and maintains the discrepancy that motivates a person to carry out further goal-consistent activities (Fishbach et al., 2006).

### Superordinate Goals Entail a Broad Scope of Context

The third characteristic of superordinate goals that can foster broad, long-term goal pursuit is a broad scope of contexts. A superordinate goal is linked to several subordinate goals across different behavioral contexts. The superordinate goal to “be healthy” cannot be fulfilled by meeting the subordinate goal to “exercise once a week” alone; it must be complemented by other subordinate goals related to diet, stress, or sleep. This broad scope of contexts can foster long-term goal pursuit in two ways. First, it follows up the process wherein focusing on a superordinate goal can lead to sustained discrepancy and thereby foster motivation and hinder compensation effects. A superordinate goal, with its broad scope of contexts, expands this process over those contexts. Second, it leads to greater flexibility in goal pursuit (see **Figure 2**).

#### Sustained Discrepancy Across Different Contexts

Compensation effects do not always remain within one behavioral context, but often spread to related contexts (e.g., indulging in an extra cigarette or deciding not to exercise that day after a healthy lunch, Nigg et al., 2009). Focusing on a superordinate goal can increase the likelihood of subsequent goal-consistent actions in various behavioral contexts and mitigate cross-context compensation effects. Achieving a subordinate goal such as “go to yoga class” signals only partial fulfillment of the goal of “be healthy.” In order to decrease this discrepancy, people may engage in goal-relevant activities in different behavioral contexts (Unsworth et al., 2013). In order to progress toward the goal of “be healthy,” the person can exercise, eat a healthy lunch, and get enough sleep. This also implies that, as long as the discrepancy is sustained, a person does not engage in cross-context compensatory behavior.

#### Increased Flexibility

A second reason that superordinate goals, with their broad scope of contexts, foster long-term goal pursuit concerns flexibility in goal pursuit. As a superordinate goal is connected to several subordinate goals, people can pursue it in multiple ways (equifinality, see Kruglanski et al., 2002). The superordinate goal of “being healthy” can be pursued by going to yoga classes, jogging, eating healthy, reducing stress, or sleeping enough. Conversely, subordinate goals can also be associated with several superordinate goals, thus promoting progress toward two or more superordinate goals at the same time. For example, the subordinate goal of “sleep 8 h a night” can contribute to both superordinate goals of “be healthy” and “be patient and kind with others”. This flexibility in goal pursuit can reduce goal conflict and can help to cope with setbacks and failures.

Goal conflicts are a major source of dissatisfaction in life and hinder goal pursuit (Carver and Scheier, 2001; Fujita et al., 2014). Goal conflicts arise when people follow multiple goals at the same time and some of those goals are mutually exclusive (e.g., Fishbach and Dhar, 2005). In order to solve the goal conflict, a person can on the one hand deprioritize, or give up one of the goals. A person who wants to indulge in chocolate can suppress the goal of eating a healthy snack and prioritize the chocolate (Kruglanski et al., 2002; Cavallo and Fitzsimons, 2012). On the other hand, instead of shielding one goal, a person could also redefine the conflicting goals in such a way that they are no longer mutually exclusive.

When focusing on a superordinate goal that allows for flexibility in goal pursuit, various options for redefining the respective subordinate goals exist and thus, various ways to solve a goal conflict are available. The subordinate goal of eating chocolate is incompatible with the subordinate goal of eating a healthy snack. However, when considering possible corresponding superordinate goals such as “enjoy life with culinary delicacies” or “lead a healthy lifestyle,” it becomes evident that there are actions that can satisfy both superordinate goals (in goal systems theory referred as “multifinality”, Kruglanski et al., 2002). Pursuing a multifinal subordinate goal that serves both linked superordinate goals offer a way to resolve the goal conflict without abandoning either of the goals. In this example, the person could eat a gourmet fruit salad instead of chocolate and thus satisfy both goals.

Besides dealing with goal conflict, successful goal pursuit also entails coping with setbacks and failures. A superordinate goal is connected to various subordinate goals (in goal systems theory referred as “equifinality”, Kruglanski et al., 2002), which allows flexibility in the means by which to approach the superordinate goal. The number of subordinate goals linked to a superordinate goal – the equifinality set – determines the amount of available choice between several subordinate goals and the range of substitutability of one subordinate goal for another (Kruglanski et al., 2002). As superordinate goals allow for flexibility in how the goal is pursued, focusing on a superordinate goal may lead to more resilience toward setbacks and failures (Robinson and Moeller, 2014). When pursuing a broad goal in the long run, it is highly probable that people will encounter setbacks and failures, such that an intended way to reach the goal is no longer possible (Rothman et al., 2004). If a certain way to reach a goal does not work out, focusing on a superordinate goal can provide a strategy to overcome this obstacle and continue pursuing the broad, long-term goal. It allows people to change the way they approach a goal without changing the goal itself. Consider a person who pursues the superordinate goal to “be healthy.” In order to achieve this goal, the person goes jogging twice a week. If she sprains an ankle, she cannot pursue the subordinate goal anymore. However, this setback doesn’t deter the person from pursuing the superordinate goal of “being healthy” as she can replace her jogging with other activities such as going to yoga classes or eating healthier. The more possibilities there are for goal pursuit, and the more likely an individual is to identify these possibilities, the better they can cope with setbacks and failures and not be immobilized by the situational limitations of the present (see also Kruglanski et al., 2002). Participants listing more subordinate goals or ways to achieve a desired attribute rated it as much easier to achieve than participants who listed only one way. The availability of more subordinate goals reduces the perceived difficulty of attaining a superordinate goal and decreases the risk of failing by bringing to mind backup strategies for reaching the superordinate goal in case of failure (Shah and Kruglanski, 2003). However, the benefits of linking several subordinate goals to a superordinate goal, and vice versa, do not mean that having more subordinate goals linked to one superordinate goal or more superordinate goals linked to one subordinate goal is better *per se*. In contrast, if the number of superordinate goals that a single subordinate goal serves increases, the perception of its instrumentality with respect to each superordinate goal decreases, a finding referred to as the dilution effect (Zhang et al., 2007). Furthermore, this dilution effect also applies to the number of subordinate goals that serve a superordinate goal: linking additional subordinate goals to a superordinate goal results in similar outcomes (Bélanger et al., 2016).

Additional support for the notion that superordinate goals make people more resilient in the face of setbacks stems from the assumption that superordinate goals are identity-based and the resulting processes. Research on self-completion theory (Wicklund and Gollwitzer, 1982) has found that people increase their effort in the face of failure if the commitment to their goal is high. If the commitment to their goal is low, people are more likely to disengage in the face of failures (Moskowitz, 2012). Focusing on a superordinate goal can increase commitment (see **Figure 2**) and thus can prevent people from disengaging in face of failure. Similarly, people are more likely to interpret difficulties in goal pursuit as implying task importance when the task at hand is congruent with their salient identity than when it is not congruent with their identity (Oyserman, 2015).

## Combining Superordinate and Subordinate Goals for Successful Goal Pursuit

Focusing on superordinate goals has many advantages for long-term goal pursuit. However, this does not mean that focusing on superordinate goals alone leads to successful goal pursuit. The outlined theoretical and empirical evidence indicates that people pursue goals in the long term with higher motivation and more consistently when they focus on both subordinate and superordinate goals than when they focus on either a subordinate or a superordinate goal alone. Subordinate goals and superordinate goals have their respective advantages and drawbacks. By combining them, people can capitalize on their respective advantages and avoid their pitfalls (Miller and Brickman, 2004; Rabinovich et al., 2009). To better understand the benefits of combining subordinate and superordinate goals, it is helpful to discuss potential drawbacks of superordinate goals and the major advantages and drawbacks of subordinate goals.

### Drawbacks of Superordinate Goals

Superordinate goals are by definition less specific than intermediate and subordinate goals. Superordinate goals are not necessarily reflected in concrete actions the way that intermediate and subordinate goals are, and are often too far removed in time to inspire immediate motivation (Locke and Latham, 1990, 2002; Bandura, 1997).

Furthermore, superordinate goals lack a specific endpoint, which could hinder goal pursuit in three ways. First, a specific endpoint allows a person to track progress in goal pursuit and to notice and address discrepancies, which foster motivation to pursue the goal (e.g., Locke and Latham, 1990; Carver and Scheier, 2001). Second, the lack of a specific endpoint makes it very hard to fulfill a superordinate goal. While the sustained discrepancy between the status quo and the desired end state can be motivating (see “Sustained Discrepancy”), it also may be too large. If people perceive a very low likelihood of goal attainment, they might get discouraged and stop pursuing the goal (Carver and Scheier, 2001; Locke and Latham, 2013). A third consequence of goals without a specific endpoint is that they are perceived as perpetually unfulfilled, and unfulfilled goals consume valuable attentional and working memory resources, which can interfere with performance in unrelated tasks that require executive function, such as anagram puzzles or dieting (Masicampo and Baumeister, 2011b). However, these detrimental interference effects disappear when people formulate specific plans for their unfulfilled goals (Masicampo and Baumeister, 2011a). This indicates that focusing on subordinate goals as well can alleviate the drawbacks of superordinate goals and facilitate goal pursuit in various ways (Locke and Latham, 1990, 2002; Bandura, 1997; Gollwitzer and Brandstätter, 1997).

### Advantages and Drawbacks of Subordinate Goals

Goals at the bottom of the goal hierarchy are concrete, specific, and have a clear start and end point. For example, the goal to “do 40 push-ups on Wednesday afternoon” clearly specifies what needs to be done in order to achieve this goal; it even specifies the time of the required action. As reaching subordinate goals requires less time than reaching superordinate goals, they can provide immediate incentives for current performance and thus boost motivation. Furthermore, goal progress and goal achievement are easy to determine with subordinate goals and therefore the frequency of feedback is increased (Sun and Frese, 2013). These qualities facilitate self-efficacy beliefs as well as task strategy development, which ultimately contributes to performance (Bandura, 1997; Sun and Frese, 2013). Additionally, with frequent feedback, people can learn from their mistakes and improve subsequent goal pursuit (Sun and Frese, 2013).

Although subordinate goals have various advantages, they can also negatively affect goal pursuit. The achievement of subordinate goals can give rise to compensatory effects that undermine goal pursuit in the long run (see **Figure 2**). For example, participants taking part in simple online games and a spelling bee task showed more complacency and decreased performance when they were exposed to subordinate goals compared to participants without subordinate goals, given that the distance to the superordinate goal was certain (Amir and Ariely, 2008).

Furthermore, focusing solely on specific, subordinate goals can lead to shortsightedness (Ordóñez et al., 2009). More specifically, subordinate goals can lead to an overly narrow focus of attention. When people focus on a goal that is narrow, they run the risk of ignoring issues that are not specified by the goal but that are important for overall goal pursuit. Managers that set specific, short-term goals, such as high half-year sales, may encourage employees to focus solely on short-term gains and to overlook potentially detrimental long-term effects for the organization (Ordóñez et al., 2009). In a similar vein, participants who had to look up a fact in the internet mostly overlooked an alternative source of information, when making a specific plan where to look for the described fact (Masicampo and Baumeister, 2012). These shortcomings of focusing on a subordinate goal or specific plan arise particularly when people face broad, long-term challenges, encounter failures and obstacles and are forced to capitalize on alternative ways to pursue their goal. This indicates that broadening perspective and focusing on an abstract, superordinate goal can offset the risks of compensation effects, shortsightedness, or narrow-mindedness.

### The Best of Both Worlds

A possible way to optimize goal pursuit and overcome the respective shortcomings of subordinate and superordinate goals is to focus on goals at different levels of abstraction, such that the functional and operational benefits of subordinate and superordinate goals can come to the fore in response to situational and task demands.

Goals at different levels of the hierarchy might not be equally beneficial or harmful across all the stages between setting a goal and achieving it. Several psychological models of behavioral change conceptualize goal pursuit and behavioral change as a process with different phases from forming a goal intention and setting a goal, to initiating an action, and finally to maintaining long-term behavior (e.g., Prochaska and DiClemente, 1982; Heckhausen and Gollwitzer, 1987; Rothman et al., 2004; Bamberg, 2013). Before starting to pursue a goal, a person has to form an intention. Based on motives, people produce wishes and desires and decide which ones they want to pursue (Heckhausen and Gollwitzer, 1987; Gollwitzer, 1990). In order to establish such a preference and to represent a wish as something that the individual feels committed to achieving which can thus motivate action, superordinate goals are crucial (Heckhausen and Gollwitzer, 1987; Oettingen, 2012). Focusing on a superordinate goal and producing a goal intention can lead to the next phase, planning. At the first stage of building a goal intention, focusing on concrete steps and procedures might be less helpful as they do not appear to generate the level of commitment necessary to actually engage in a first behavior (Freitas et al., 2004). However, they are of great help in the next phase.

Any attempt to motivate goal pursuit via superordinate goals needs to make sure that people are either already aware of their behavioral options or that they are provided with the necessary action knowledge. Otherwise superordinate goals can be too abstract and disconnected from actual behavior and thus not provide enough information about goal attainment (Bandura, 1997; Moussaoui and Desrichard, 2016). In this case, superordinate goals would imply just imagining a desired future outcome unrelated to the present situation. Simply thinking about a positive outcome can have adverse effects on subsequent goal pursuit, as it induces a feeling of accomplishment and thus satisfies desire and reduces discrepancy (e.g., Oettingen, 2012; Baumeister et al., 2016). Experimental findings in various contexts such as academic and career success, relationships and health support this (e.g., Oettingen, 2000; Oettingen and Mayer, 2002). In order to transition from a goal intention to planning and acting it is crucial that people are aware that they have not yet fulfilled their wish and that they need to take steps to achieve their desired outcome (Oettingen, 2012). Thus, after setting a superordinate goal, the next task is to select a new behavioral strategy and form a goal intention. Accordingly, the goal setter should address questions of when and where to start acting, how to act, and how long to act. Subordinate goals play a crucial and central role in this phase (Locke and Latham, 2013). Furthermore, at this stage, after having made a commitment, focusing on a superordinate goal could be a functional strategy to support one’s goal pursuit. For example, reflecting on the reasons why a person should pursue an already chosen goal – and thus activating a superordinate goal – can lead to information processing in defense of the chosen goal and to an effortful top-down approach which can strengthen the commitment to the goal and foster goal pursuit. This effect takes place when a person has sufficient cognitive capacity to engage in the effortful reflection on the “why” (Nenkov and Gollwitzer, 2012; Wieber et al., 2014).

After setting goals, the phase in which an action is initiated begins (Prochaska and DiClemente, 1982; Heckhausen and Gollwitzer, 1987; Bamberg, 2013). Goal-setting theory has shown that, especially when initiating an action, subordinate goals boost motivation and foster goal pursuit more than do abstract, vague goals (Locke and Latham, 2013). Recent research also emphasizes possible advantages of less specificity and thus also a possible positive effect of superordinate goals during this action initiation phase (Wallace and Etkin, 2017). Specificity of goals – that is, having a clear endpoint – increases motivation as a function of goal proximity. Several streams of research (e.g., goal looms larger effect, Förster et al., 1998; goal gradient hypothesis, Hull, 1932; Kivetz et al., 2006) state that motivation increases with proximity to the endpoint. To illustrate, a dieter with the goal to lose 10 pounds is more motivated to lose an additional pound when he has already lost 8 pounds than when he just started, as he sees losing the 9th pound as more impactful than losing the first. However, goals without a specific endpoint lack that motivation boost. It can be argued that in the absence of a specific endpoint the initial state acts as reference point. Adopting an initial state as a reference value could lead to greater motivation at the beginning of goal pursuit; for example, after losing the first pound. In this case, the dieter is closer to the initial state than the desired end state and thus adopting the initial state as reference point can boost motivation (Wallace and Etkin, 2017).

Long-term behavior change and the formation of habits not only require behavior initiation, but also maintenance and extended repetition of that behavior – the next phase in goal pursuit (Rothman et al., 2004). However, people who successfully initiate an action more often than not fail to sustain the behavior over time (Miller and Brickman, 2004). Focusing on superordinate goals can help to overcome the challenges in this phase; they help to sustain motivation after achieving subordinate goals and over the long term (see “Sustained Discrepancy”), to resist distractions and temptations (see “Heightened Importance”), to resolve goal conflicts and to be resilient in the face of setbacks and failures (see “Increased Flexibility”). In the latter case, when not everything works out as planned and people encounter difficulties, superordinate goals help by allowing people to change how they pursue the goal. However, if a person does not want to give up on a subordinate goal, but rather to try again, a focus on subordinate goals might be helpful. In circumstances of difficulty or stress, a subordinate goal that specifies what to do next and how to do it might foster motivation and help a person to overcome the challenge. This is in line with Action Identification Theory that states that when people face difficulties, they tend to shift to more concrete levels to focus on how to carry out the action and to connect them with the current environment (Vallacher and Wegner, 1987; Watkins, 2011) and with recent research that shows that people who experience failure benefit from focusing on subordinate goals (Houser-Marko and Sheldon, 2008). This implies that a focus on both subordinate and superordinate goals is helpful in this phase, which is further demonstrated by research on proximal and distal goals. This research shows that people who focus on both types of goals perform better than those who focus on distal goals alone (Bandura, 1997; Latham and Seijts, 1999; Steel and König, 2006). The motivational effect of combining superordinate goals with subordinate goals is also reflected in research on possible selves. Thinking about how one wants to be in the future (i.e., focusing on a superordinate goal) is more motivating when the possible self is linked to concrete strategies and actions (Oyserman and James, 2011). For example, eighth graders improve grades, spend more time on homework, show increased participation in class, and are referred to summer school less when academic possible selves are linked to concrete strategies for attainment (Oyserman et al., 2004). Furthermore, students feel more academically motivated when they consider specific action plans linked to their future self, and less academically motivated when they focus on the outcomes of the future self (Peetz et al., 2009, study 2).

In order to change a behavior in the long term and sustainably, it helps if the behavior becomes habitual (Lally and Gardner, 2013). The focus on superordinate goals help the goal setter to repeat a behavior over a longer period of time and thus lays the foundation for habit formation. However, for a behavior to become habitual, it must become an automatic response to environmental cues and may occur in the absence of awareness, conscious control, mental effort and deliberation – that is, in the absence of conscious goal pursuit (Lally and Gardner, 2013). Implementation intentions – strategies that help people to plan when, where and how they will strive for a goal and thus tie a behavior to a specific situation (e.g., “if I encounter situation X, I will perform action Z”) – are shown to foster goal-relevant behaviors in the absence of cognitive control as they delegate control over the initiation of a goal-relevant behavior to a specified situational cue (Sheeran et al., 2005; Wieber et al., 2014). While focusing on a superordinate goal before forming implementation intentions has been shown to foster goal-relevant behavior (e.g., Adriaanse et al., 2010; Kappes and Oettingen, 2014), focusing on a superordinate goal at the same time as exerting implementation intentions could have detrimental effects on goal pursuit, as focusing deliberately on the “why” of goal pursuit could hinder the automatic response initiation that implementation intentions are supposed to foster (Wieber et al., 2014). This highlights the relevance of temporal order in the interplay of subordinate and superordinate goals.

The suitability of focusing on a subordinate and/or superordinate goal also depends on task demands and personal skill level. According to Action Identification Theory (Vallacher and Wegner, 1987), people are effective in performing a behavior when the task difficulty matches the cognitive representation of the task: When a task or behavior is difficult, it is better to identify an action on a lower level, thereby focusing on the “how”. In contrast, when a behavior is easy, a higher-level identification leads to better performance (Vallacher and Wegner, 1987). Recent research confirms this prediction and shows that writing about how to pursue a goal leads to better performance for individuals with lower domain-specific skills, and writing about why to pursue a goal leads to better performance for individuals with higher domain-specific skills (Ferguson and Sheldon, 2010). This highlights the necessity of matching a focus on a subordinate and/or superordinate goal to a person’s skill level and task difficulty, in order to allow a person to address the most salient barriers to successful goal pursuit (Ferguson and Sheldon, 2010).

## Summary

The theoretical and empirical evidence outlined in this article suggests that people are more likely to successfully pursue their goals over the long run when they focus on both superordinate and subordinate goals. While the received opinion in the field of goal research is that focusing on concrete and specific subordinate goals is the best way to achieve one’s goals, there is compelling theoretical and empirical evidence for the strengths of superordinate goals. Specifically, focusing on superordinate goals can foster broad, long-term goal pursuit through multiple processes: increased long-term motivation and effort while pursuing the goal; inhibition of competing goals, distractions and temptations; stimulation of behavioral consistency, as it can inhibit compensation effects; strengthening of resilience, as it aids in coping with obstacles, failures, and setbacks and helps to resolve goal conflicts due to more flexibility in goal pursuit.

All these potential benefits are assumed to come into effect in particular when superordinate goals are combined with subordinate goals. Combining goals at different hierarchical levels is a promising approach for successful goal pursuit, as the benefits of superordinate and subordinate goals come to the fore while the disadvantages are balanced out. An integral part of understanding how goals operate is to understand how goals at different hierarchical levels influence goal pursuit and interact with each other.

## Recommendations for Future Research

While the advantages of focusing on subordinate goals are widely supported in the literature, the benefits of also focusing on superordinate goals are insufficiently studied. Long-term studies and a comparison between distinct effects of subordinate goals and superordinate goals, as well as their effect in combination, are needed.

Another avenue for future research is the dynamic interplay of superordinate and subordinate goals. First, future research could address questions such as how subordinate goal performance and feedback influence the commitment to the end goal, or how focusing on a superordinate goal influences the setting of subsequent subordinate goals. Second, future research could address the role that superordinate and subordinate goals play across the various phases of goal pursuit (e.g., action phase model, Heckhausen and Gollwitzer, 1987; transtheoretical model, Prochaska and DiClemente, 1982) and explore the ways in which switching between superordinate and subordinate goals could facilitate goal pursuit, especially in the long term.

Finally, the present article focuses on conscious choice and guidance of behavior. Behind this lies the idea that one actively sets oneself a goal and pursues it. However, much of modern psychology has recognized that goal pursuit is also rooted in processes outside of conscious control (Bargh et al., 2001): goals not only become activated by conscious choice but also unconsciously (see for example bottom-up goal activation, Shah and Kruglanski, 2003). This raises the question of whether it is possible to focus specifically on a subordinate or superordinate goal if they are connected to each other, or if the focus on the one automatically – outside of one’s awareness – activates connected goals at another hierarchy level. It leaves open the question of to what extent the distinct processes and advantages of focusing on a subordinate and/or superordinate goal can be observed in isolation and thus whether a clear distinction between the activation and resulting processes of subordinate and superordinate goals is feasible (also with regard to future experiments). There is initial evidence that an additional activation of superordinate goals – even if unconsciously – can trigger processes that go beyond the activation of only one subordinate goal (and the potentially triggered bottom-up activation of a superordinate goal). Fishbach et al. (2006) conducted four studies in which participants made two successive decisions. Participants acted more goal-consistent when a superordinate goal had unconsciously been primed (e.g., through an ostensibly unrelated scrambled sentence task with words related to a superordinate goal, or through pictures related to a superordinate goal attached to the clipboard of the survey) than when they focused solely on the subordinate goal (Fishbach et al., 2006). Future experimental research is needed to address optimal ways to activate – either consciously or unconsciously – goals at an appropriate and beneficial level of abstraction.

## Conclusion

Pursuing goals in the long run is crucial to addressing challenges on a personal level (e.g., eating a healthy diet), on an organizational level (e.g., lasting employee effort), and on a societal level (e.g., consistent environmentally friendly behavior). Expanding goal-setting theory to consider goals at different levels of abstraction promises to enrich the understanding of how goals operate and to widen the knowledge about the determinants of effective goal setting and goal striving. This understanding and knowledge provides a basis for interventions that can help people to select and pursue their goals successfully in the long run and thereby achieve sustainable behavioral change. While much remains to be discovered, the research outlined here suggests that people pursue goals in the long term better when they focus on both subordinate and superordinate goals as compared to when they focus on either subordinate or superordinate goals alone. Thereby it provides a basis for future empirical research.

## Author Contributions

BH, AB, and CM contributed to the main conceptual ideas and the writing of the manuscript.

## Conflict of Interest Statement

The authors declare that the research was conducted in the absence of any commercial or financial relationships that could be construed as a potential conflict of interest.
